# In Vitro Evaluation of the Effects of Multipoint Hyaluronic Acid‐Based Intradermal Fillers on Skin Quality Using a Novel 3D Reconstructed Skin Aging Model

**DOI:** 10.1111/jocd.70362

**Published:** 2025-09-11

**Authors:** Marion Albouy, Morgan Dos Santos, Kilian Laho, Denis Couchourel, Frédéric Tranchepain

**Affiliations:** ^1^ Laboratoires VIVACY France; ^2^ LabSkin Creations Lyon France

**Keywords:** 3D skin model, aesthetic medicine, hyaluronic acid, regenerative aesthetic, skin quality, skin regeneration, tissue engineering

## Abstract

**Background:**

Superficial injection of hyaluronic acid (HA)‐based gels is a widely used method to restore skin quality and achieve a more youthful appearance. While the clinical benefits of such procedures are well established, their biological mechanisms of action remain poorly understood.

**Objective:**

This study aimed to evaluate the effectiveness of two cross‐linked HA gels (IPN‐12.5 and HA‐X12) on skin rejuvenation, focusing on skin hydration and extracellular matrix quality. The evaluation was conducted using an innovative skin model that closely mimics native human skin.

**Material and Methods:**

A bioengineered scaffold composed of collagen, glycosaminoglycans, and chitosan was developed and seeded with aged human fibroblasts. This seeded scaffold has the ability to allow cellular migration, proliferation, and neo‐synthesize its own extracellular matrix. Normal human keratinocytes were subsequently seeded on top of the newly formed dermal equivalent, thereby simulating the complex interactions that occur between the epidermis and dermis. Multipoint intradermal injections of the test products were administrated using a multipoint technique during the reconstruction protocol.

**Results:**

Both products significantly decreased transepidermal water loss (TEWL), though histomorphological differences were observed. The IPN‐12.5‐hydrogel‐injected models showed enhanced barrier integrity, water retention, epidermal renewal, and skin elasticity markers compared to the controls and HA‐X12.

**Conclusion:**

IPN‐12.5 hydrogel significantly improved hydration and elasticity‐related skin markers when compared to a similar product (HA‐X12). Despite similar clinical indications and concentrations, the products exhibited different biological effects, potentially due to differences in cross‐linking technologies or HA molecular weights. These factors are likely to influence clinical outcomes achieved with the two formulations.

## 1 Introduction

Superficial injections are currently gaining popularity in aesthetic medicine due to their minimally invasive nature. Although hyaluronic acid (HA)‐based fillers tend to be mainly used in protocols intended specifically for correcting facial wrinkles and volume deficiencies through deep injections, targeting the dermis with HA‐based gels is a validated method for restoring skin quality and a youthful appearance [1]. Superficial injection‐based protocols aim to increase hydration and reconstruct an optimal physiological environment for fibroblasts and fibroblastic stimulation, thereby enhancing their cellular activity and synthesis of extracellular matrix structural molecules, like collagen and elastin. Both cross‐linked and non‐cross‐linked HA have been used to trigger those biological effects in the skin to restore its initial youthful properties. Aside from the differences in the characteristics of the products used, one of the key points that differentiates them from more conventional volumizing HA‐based gels is the method of their injection. This consists of micro‐dosed repeated injections of bioactive products that are precisely placed targeting the dermal region. If this technique is performed correctly, this type of treatment is capable of triggering extreme hydration and good improvement of the overall appearance of the treated skin, which can be useful to revert typical aging signs, particularly in the face or neck [1], However, HA‐based gels dedicated for superficial injections have essentially been studied through clinical evaluation, and very little is known about their exact mechanism(s) of action.

Skin aging and dehydration are often correlated. Aging causes the skin to become thinner, making it susceptible to trans‐epidermal water loss and subsequent dehydration. Control of skin water content is a critical element for maintaining skin health, and this parameter is dependent on two main physiological features: the presence of aquaporin‐3 (AQP3) and the skin barrier composition [2]. Sougrat et al. [3] first reported that AQP3 distribution in the epidermis was consistent with epidermal water distribution and parallels the steep water gradient at the junction between the *stratum granulosum* and the *stratum corneum* (SC). In the viable epidermis, AQP3 acts as a channel and allows the passage of water through cellular membranes to form a short circuit between the base of the epidermis and the SC to maintain a constant water level, essential for cells to function healthily. It has been demonstrated that AQP3 expression decreases with aging and that it is associated with defective osmotic equilibrium in the epidermis, leading to skin dryness [4]. The SC, which contains non‐nucleated keratinocytes (corneocytes), is the most differentiated epidermal layer and provides the barrier function to the skin, regulating skin permeability and aiding in preventing transepidermal water loss (TEWL) in youthful skin. Furthermore, the natural moisturizing factor (NMF) is a component of corneocytes that has water‐binding properties and is derived from the proteolytic breakdown of the filaggrin protein [5]. Indeed, intracellular filaggrin undergoes degradation into constituent amino acids as corneocyte differentiation progresses, which, along with their post‐translation modified derivatives, form major constituents of the epidermal NMF. The regulation of this process directly depends on the hydration status of the skin, and dysregulation or reduced levels of NMF may be associated with several skin conditions, including atopic dermatitis, ichthyosis, xerosis, and psoriasis [6].

HA is an important regulator of skin hydration that helps in maintaining skin structural integrity and its barrier function due to its water‐binding properties. It also interacts with keratinocytes to regulate lipid synthesis and keratinocyte differentiation [6]. Additionally, it has been demonstrated that the binding of endogenous and exogenous hyaluronic acid to its main ubiquitous surface receptor, CD44, improves permeability and barrier homeostasis by stimulating keratinocyte differentiation and lamellar body formation in the SC [7]. More globally, several studies have revealed the key role of CD44 in promoting angiogenesis and tissue repair by mediating cellular migration and interaction with the extracellular matrix (ECM) during the wound healing process [8].

All the previously described epidermal targets of interest are constantly expressed at fluctuating levels in differentiated skin layers despite the epidermal perpetual renewal throughout life due to the existence of a stem cell reservoir, which takes place in a very specific region located near the epidermal basal membrane. Collagen XVII is an important transmembrane protein whose role is to mediate interaction of stem cells with their surrounding cells and matrix. Indeed, collagen XVII expression at the dermal epidermal junction (DEJ) level is essential for stem cell function and the prevention of skin aging [9]. Additionally, Laminin‐332 is also a key component of the basal membrane, providing stable attachment of the epidermis to the dermis and thus plays an important role in skin homeostasis and epidermal basal layer organization [10].

However, the epidermis is not the only compartment strongly impacted by the intrinsic and extrinsic aging process. The dermal compartment's thickness, collagen, and elastic fibers tend to degrade dramatically [11]. Type I collagen undergoes organizational and structural changes leading to its reduced strength, mainly due to high levels of degradation and fragmentation without being replenished by the dermal fibroblasts. Additionally, EMILIN‐1 (elastin microfibril interface‐located protein 1) is a protein highly linked to changes in the elastic fibers structure and stability of their formation [12].

Injectable hydrogels manufacturers tend to use in vitro models that have the ability to demonstrate the biological responses of cutaneous cells or tissues after injections in different anatomical planes. At Vivacy Laboratory, we chose to focus on a reconstructed skin model because, despite missing some cell types, the presence of a highly standardized protocol containing the primary skin cells has strong advantages. We believe that the main advantage is that it reproduces a metabolically active and homeostatic biological system able to respond to external stimuli. This model enables the monitoring of changes in extracellular and epidermal components. Other manufacturers have recently chosen to work on skin explants to demonstrate the effectiveness of their product [13], even going as far as developing their own in‐house model [14], to enable them to adapt their culture conditions depending on the tested product.

Nonetheless, even under optimized conditions, signs of tissue deterioration, like epidermal detachment or fragmentation of extracellular matrix content, tend to appear after 5 days in skin explants. Moreover, levels of expression of targeted proteins gradually decreased over time even without any treatment, hence posing a serious constraint on the duration for which skin explants can be kept in culture and the plausibility of interpreting any observed biological responses. However, this very limited culture time prevents the recommended treatment protocols from being carried out. Indeed, 2–3 injection sessions are commonly recommended for treatments aimed at addressing skin quality, a protocol that is not feasible when using skin explants [13]. These limitations led us to favor a study on a skin model rather than on a skin explant for the evaluation of our IPN‐12.5 formulation.

The aim of this study was to compare the impact of two different cross‐linked hyaluronic acid‐based gel formulations (IPN‐12.5 and HA‐X 12) on their ability to influence the various skin elements, as described above, that have been linked to the process of skin aging. In this study, we used an innovative and versatile three‐dimensional (3D) full‐thickness skin equivalent (SE) model mimicking the skin aging process to explore the biological effects of these HA‐based products.

## 2 Materials and Methods

### 2.1 Ethical Considerations and Human Cutaneous Cell Isolation

Human skin tissues were collected from surgical discard from anonymous healthy donors. Surgical residues were anonymized, and written informed consent was obtained from patients in accordance with the ethical guidelines from Lyon University Hospital (Hospice Civils de Lyon) and approved by the ethical committee of the Hospices Civils de Lyon according to the principles of the Declaration of Helsinki and Article L. 1243–4 of the French Public Health Code. All the samples used in this study belong to a collection of human skin samples declared to the French Research Ministry (Declaration no. DC‐2020‐4346 delivered to LabSkin Creations, Lyon, France).

Primary cultures of normal human fibroblasts and keratinocytes were established from healthy skin biopsies obtained from an adult Caucasian donor (> 40 years old).

### 2.2 Chemicals/Products

IPN‐12.5 is an innovative monophasic cross‐linked HA‐based gel (12.5 mg/g) and is indicated to improve hydration and cutaneous elasticity of the face, neck, and décolleté. IPN‐12.5 is classically injected into the mid to deep dermis. IPN‐Like technology, developed by Laboratoire Vivacy, is a unique cross‐linking process that helps to define product viscoelastic properties. HA‐X‐12 is a competing HA‐based cross‐linked injectable gel that is also indicated to fill superficial cutaneous creases and to improve fine lines and skin quality attributes (such as hydration and elasticity). This product was chosen as a comparator because, in addition to having an HA concentration almost identical to IPN‐12.5 (12 mg/g), it has very similar rheological characteristics. To the best of our knowledge, this is the first report of a full‐thickness skin model used for the evaluation of an injectable device with repeated treatment sessions.

### 2.3 3D Aging Full‐Thickness Reconstructed Skin

This reconstructed skin model, based on a porous scaffold made of a mix of bioinspired materials, has been used for several years by several industrial and academic teams to study extremely diverse biological processes. Indeed, the fact that it behaves so closely to native cutaneous tissue has encouraged its use in multiple areas of skin biology.

In 2005, Dos Santos et al. [15] explored its behavior in long‐term culture conditions to mimic chronological aging; they concluded that the major morphological and ultrastructural dermal and epidermal modifications observed in both the extended culture skin equivalent model and skin biopsies from old donors validated the relevance of this model for studying chronological aging, understanding, and elucidating age‐related modifications of basic skin biological processes. Moreover, they confirmed that this model provided a unique tool for identifying new targeted molecules aimed at improving the appearance of aging skin. This model has also been widely used to study the wound healing process [16] and evaluate skin repair and antiaging treatments [17]. To the best of our knowledge, this is the first time it has been used for the evaluation of injectable medical devices. The researchers are confident that the chosen model is the most judicious among all existing in vitro/ex vivo models and that the product injection protocols that have been developed in the context of this study are robust and as reproducible and clinically relevant as possible.

In practice, this 3D full‐thickness reconstructed skin model was obtained by culturing normal human dermal fibroblasts (NHDF), extracted from a 42‐year‐old donor, in a scaffold made of collagen, glycosaminoglycans, and chitosan (LabSkin matriX) for 28 days under optimized cell culture conditions for ECM neo‐synthesis. Normal human epidermal keratinocytes (NHEK) were then seeded on top of the dermal equivalent constructs and raised to the air/liquid interface to allow the formation of the epidermal compartment [18]. Intradermal injections were conducted manually at a 45° angle from the surface of the 3D construct to ensure an identical penetration depth, using a 30G^1/8^ needle. A total of 15 points of intradermal injections were conducted at each treatment session with a total volume injected of 100 μL in each skin model. Aging SE constructs were treated three times during the culture, and injections were performed by the same researcher under the same aseptic conditions described above. The first injection was made during dermal reconstruction (day 18), the second after NHEK seeding (day 32), and the third one after the SE was raised to the air–liquid interface (day 40).

3D aging SE samples harvested at day 54 of total cell culture were immediately fixed in neutral buffered formalin 4% (Diapath) for 24 h and embedded in paraffin or in Optimal Cutting Temperature (OCT) compound and frozen at −80°C for histological and immunohistological analysis, respectively. For each cell culture condition and analysis, 3D SEs were produced in triplicate. It is known that the mechanical trauma caused by the needles during injections themselves can exhibit a stimulating effect on the skin. To eliminate this potential bias, the untreated (UT) samples (controls) were reconstructed skin injected several times with phosphate saline buffer using an identical needle and a similar injection protocol to that used for injecting the HA‐based products. UT samples were treated in the same experimental conditions as the HA‐based fillers conditions.

### 2.4 Measured Parameters and Procedures

The epidermal formation and dermal extracellular matrix synthesis were assessed by means of their histological structure and TEWL. Deposition of extracellular matrix components was assessed by type 1 collagen deposition and EMILIN‐1 expression. Epidermal basement membrane remodeling was evaluated by the expression of laminin‐332 and type XVII collagen, as well as the number of proliferative Ki‐67‐positive cells. Finally, epidermal homeostasis, late differentiation (filaggrin), lipid synthesis (ceramides), cellular cohesion (CD44), and tissue hydration (AQP3) were examined to evaluate terminal differentiation and cellular cohesion.

### 2.5 Transepidermal Water Loss (TEWL) Analysis

TEWL was obtained using a VapoMeter (Delfin Technologies, Finland) with a closed measuring chamber and standard tip. For the measurement, the reconstructed skin was placed with the SC face down on the vertically held VapoMeter probe. Between each measurement, with the sample placed on an absorbent paper covered with a petri dish lid to prevent it from drying out, the VapoMeter probe was ventilated to allow the removal of accumulated moisture. Three measurements were taken for each reconstructed skin sample.

### 2.6 Histological Analysis

The samples' global cutaneous structure was assessed using hematoxylin–Phloxine–Saffron (HPS) staining. For each condition, 5 μm paraffin sections were sliced. Following dewaxing and rehydration, the samples were stained with HPS. After rinsing, the sections were dehydrated before mounting on slides with a hydrophobic mounting medium.

### 2.7 Immunohistological Analysis

For immunofluorescence on paraffin sections, nonspecific binding was blocked in phosphate buffered saline (PBS) containing 4% bovine serum albumin (BSA) following heat‐mediated antigen retrieval treatment. Sections were then incubated with the primary antibodies of interest diluted in PBS/BSA 4% overnight at room temperature. After incubation for 1 h with an AlexaFluor‐568‐conjugated anti‐mouse/rabbit secondary antibody (Molecular Probes, Invitrogen), nuclear counterstaining was carried out using 4′, 6‐diamidino‐2‐phenylindole (DAPI). For immunohistochemistry on paraffin sections, nonspecific binding was blocked in PBS containing 4% BSA after heat‐mediated antigen retrieval treatment. Sections were then incubated with the primary antibodies of interest diluted in PBS/BSA 4% overnight at room temperature. After 1 h of incubation with an anti‐mouse/rabbit conjugated with horseradish peroxidase (HRP) secondary antibody (Dako EnVision+, HRP) followed by the addition of 3,3′ diaminobenzine (DAB) + substrate solution addition, tissue samples were counterstained by immersion in 25% Harris Hematoxylin counterstaining solution. As a negative control, the primary antibody was replaced by the corresponding IgG class.

Antibodies directed against CD44 and Laminin‐332 were purchased from Santa Cruz (California, USA); those directed against AQP3, collagen XVII, and EMILINE‐1 were purchased from Abcam (Cambridge, Royaume Unis). Concerning the antibodies used for the detection of Ceramides, Filaggrin, Ki67, and Collagen I, they were respectively purchased from Glycobiotech (Hamburg, Germany), Genetex (Irvine, California, USA), DakoCytomation (Glostrup, Denmark), and NOVOTEC (Lyon, France).

### 2.8 Microscopic Image Acquisition and Analysis

Specimens stained in HPS were examined using an Axioskop 2 Plus optical microscope (Zeiss), and images were captured using a DS‐Ri1 CCD camera (Nikon) and NIS‐Elements software (Nikon). Sixteen‐bit images were saved in an uncompressed tagged image file format (tiff). Nine representative images were captured for each condition in the same manner.

### 2.9 Cutaneous Markers Quantification

Positively red‐stained tissue areas were automatically detected and segmented from other pixels. Images were then converted to binary images, treated by mathematical morphology, and sieved to isolate the regions of interest. The surface area of interest was measured automatically. Data were normalized by the dermal–epidermal junction or stratum corneum length for epidermal markers and by the dermal area for dermal markers. Data are expressed in percentage of density.

### 2.10 Epidermal Thickness Measurement

Epidermal thickness was obtained with a Euclidean distance map. Pixels corresponding to the epidermis were selected from other pixels. Images were converted to 8‐bit binary images. Images corresponding to the area of interest were converted to a 16‐bit distance map. For each epidermis pixel (nonzero) in the distance map binary image, a value equal to its distance from the nearest background pixel (zero) was assigned. The epidermal basal line was selected and then applied on the distance map. The mean intensity of the basal line corresponded to the mean distance between the basal line and the SC. Data are expressed in μm.

### 2.11 Statistical Analysis

For all data, the statistical significance was assessed using a one‐way Student's test, and statistically significant differences are indicated by asterisks as follows: *p < 0.05, **p < 0.01, and ***p < 0.001.

## 3 Results

**FIGURE 1:**
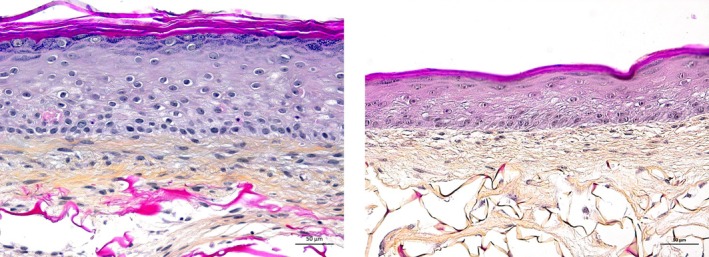
Representative images of hematoxylin–phloxin–saffron‐stained performed on obtained skin samples reconstructed with fibroblasts extracted from a young (left) or aged donor > 40 years (right).

Figure 1 shows the morphological analysis by hematoxylin, phloxin, and saffron (HPS) staining of a SE model engineered with cells derived from young and old donors. In untreated samples, SE generated from young fibroblasts was superior to SE from aged fibroblasts (Figure 1) in all histological characteristics, including living epidermal layers, terminal differentiation, and dermal extracellular matrix that were present in abundance. This first element of comparison allowed us to validate the basal “aged” phenotype of samples in our study and to be more confident in the relevance and predictability of our model.

### 3.1 Effect of IPN‐12.5 on Epidermal Formation and Dermal Extracellular Matrix Synthesis on Aging SE Treated Model

**FIGURE 2:**
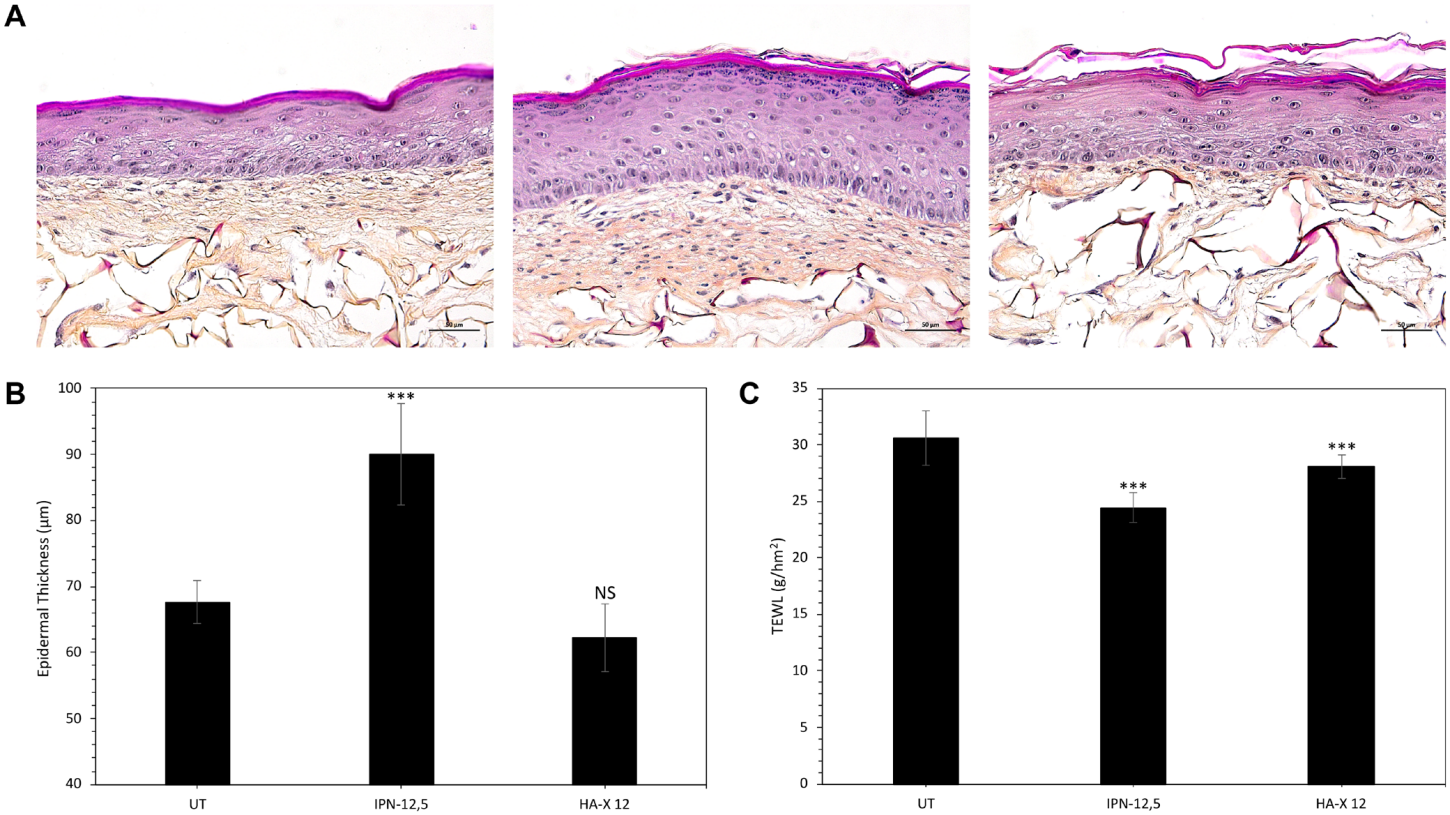
Study of global morphology and Transepidermal Water Loss (TEWL) values of obtained reconstructed skin samples intradermally injected or not with hydrogels of interest. (A) Representative images of hematoxylin–phloxin–saffron‐stained reconstructed skin. (B) Quantification of mean epidermal thickness for each condition of treatment. Analysis was performed using three reconstructed models per condition of treatment, and three representative pictures were taken by sample. (C) Mean TEWL values measured on reconstructed skin samples for each condition of treatment. Student's test: *p < 0.05, **p < 0.01, ***p < 0.001.

Figure 2A shows the morphological analysis of hematoxylin, phloxin, and saffron (HPS)‐stained 54‐day SE model engineered with cells derived from the donors. Both IPN‐12.5 and HA‐X12 treated 54‐day aged SE model were associated with significant changes at the epidermal and dermal layers (including extracellular matrix abundance) compared to the 54‐day SE‐untreated aged control (Figure 2A).

At the epidermal level, IPN‐12.5‐injected SE models displayed a significant increase in the thickness of the epidermis with a higher degree of maturation of the upper layers compared to the untreated control. The epidermal compartment appeared multilayered, stratified, and well differentiated with 8–10 supra‐basal cell layers, while the untreated control continued to exhibit 2–3 layers of cells only. Image analysis quantification confirmed that the epidermal compartment was significantly thicker in IPN‐12.5‐injected SE samples than the UT control (Figure 2B). However, HA‐X 12‐treated 54‐day SE aged samples did not show a significant change in the epidermal compartment compared to the UT control (Figure 2A,B).

At the dermal level, IPN‐12.5 stimulated the colonization of the whole dermal substrate by the aged NHDF, resulting in abundant neo‐synthesis of ECM that filled the porous structure of the scaffold compared to the aged untreated control. The dermal layer and underlying DEJ (often referred to as “papillary‐like dermis”) appeared significantly thicker and denser. In contrast, the HA‐X 12‐treated aged condition showed a lower density of the ECM components, displayed a lower number of fibroblast cells, and a thinner “papillary‐like dermis” compared to both the untreated aged control and the IPN‐12.5‐treated SE constructs.

The barrier functionality of the 54‐day aged constructs was determined by detailed examination of tissue histological organization. Stable measurements revealed a significant decrease in mean TEWL values in both IPN‐12.5 and HA‐X 12‐treated conditions compared to the UT control (Figure 2C). Mean TEWL values were decreased by 20% (*p* < 0.001) and 8% (*p* < 0.01) following IPN‐12.5 and HA‐X 12 injections, respectively.

### 3.2 Effect of IPN‐12.5 on Deposition of Extracellular Matrix Components in the Aging Reconstructed Dermis

**FIGURE 3:**
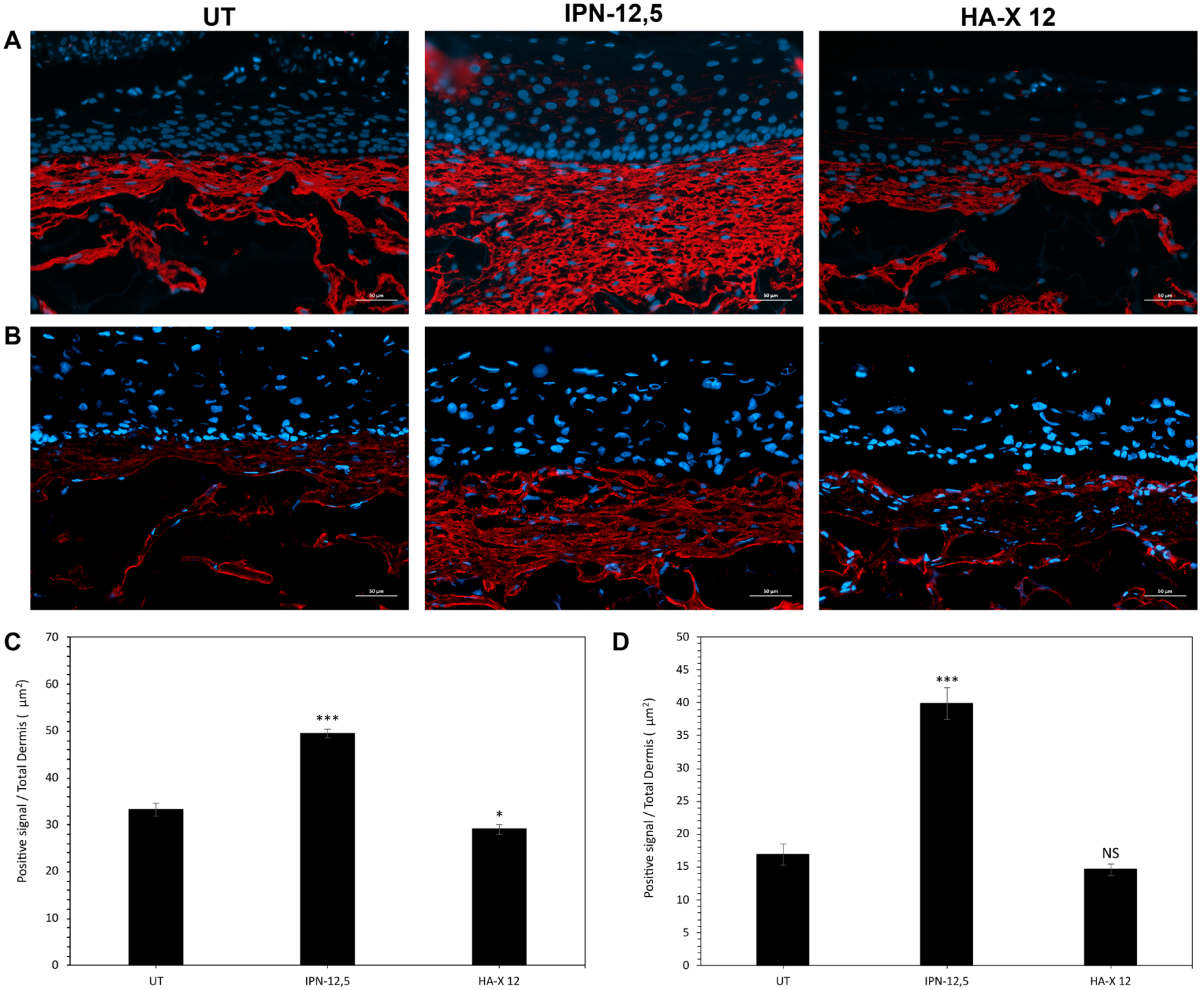
Effects of the two evaluated hyaluronic acid‐based hydrogels on type I Collagen and EMILIN‐1 expression in reconstructed skins. (A) Immunostaining of type I Collagen in the obtained dermis. (B) Immunostaining of EMILIN‐1 I in the obtained dermis. Quantification of (C) Type I collagen and (D) EMILIN‐1 expression levels. Analysis was performed using three reconstructed models per condition of treatment and three representative pictures were taken by sample. For each picture, total positives red pixels were rationalized by dermal area. Student's test: *p < 0.05, **p < 0.01, ***p < 0.001.

To further explore possible mechanisms responsible for dermal deposition‐induced modulation by intradermal IPN‐12.5 and HA‐X 12, we investigated the expression of several aging dermal‐related markers in HPS‐stained samples (Figure 3). Representative microscopic imaging revealed regular and consistent deposition of type I collagen throughout the dermal equivalent in the UT control. In IPN‐12.5‐treated samples, the synthesis of this matrix protein was highly enhanced, suggesting promotion of ECM organization and stability, while the HA‐X 12‐injected condition did not show any effect on collagen expression. Additionally, compared to the UT control, there was a significant increase in the expression of EMILIN‐1 with IPN‐12.5 only (Figure 3B).

Quantitative evaluation by image analysis of ECM components in SE confirmed these immunohistochemical observations and demonstrated a significant increase in type I collagen (+48%; *p* < 0.001, Figure 3C) deposition and EMILIN‐1 (+136%; *p* < 0.001, Figure 3D) expression in the IPN‐12.5‐treated SE compared to the UT control. In contrast, there was a significant decrease in both type I collagen and EMILIN‐1 in HA‐X 12‐injected conditions by 12% and 13%, respectively, compared to the UT aged control (Figure 3C,D).

### 3.3 Effect of IPN‐12.5 on Epidermal Basement Membrane Remodeling

**FIGURE 4:**
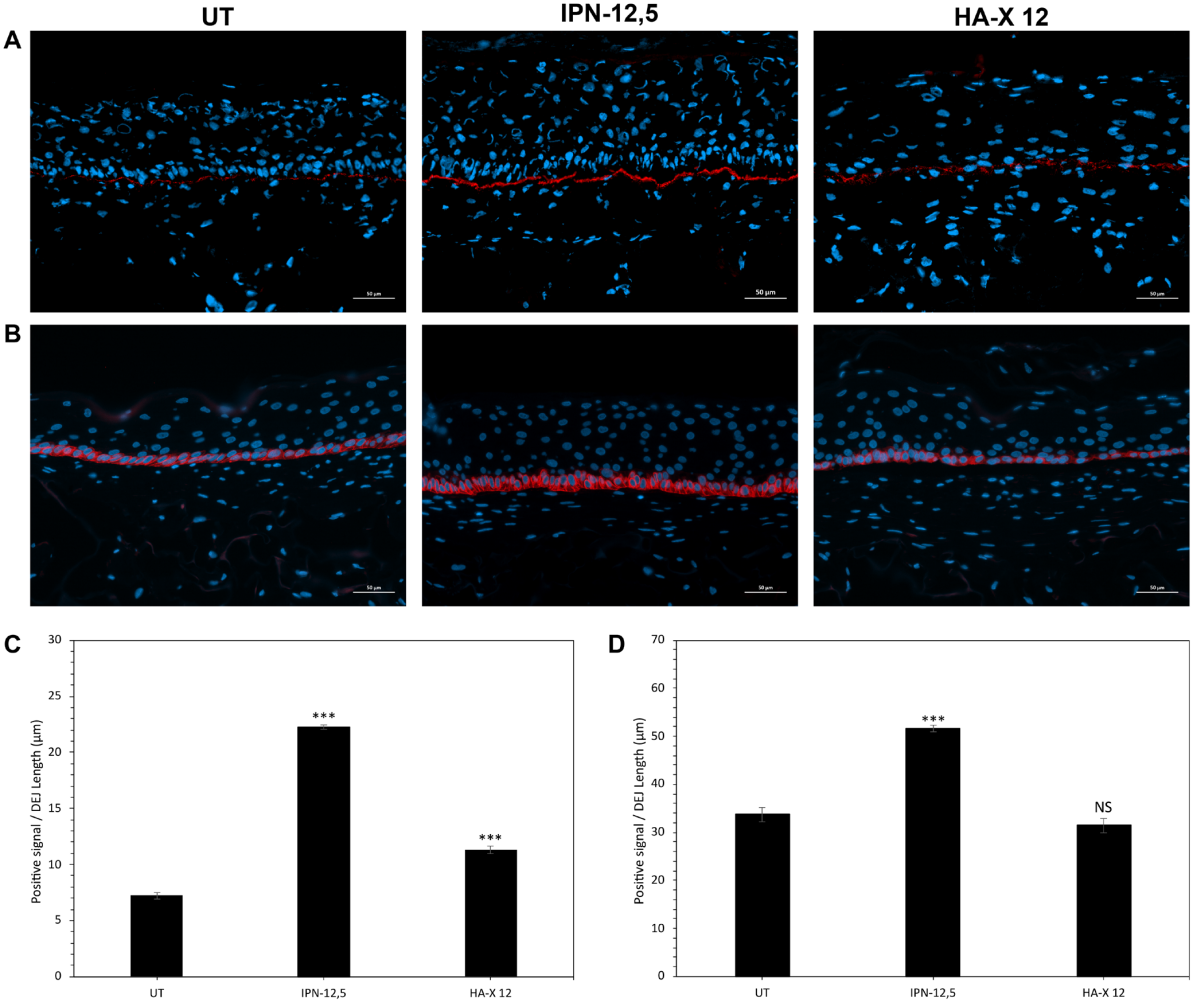
Effects of the two evaluated hyaluronic acid‐based hydrogels on Laminin‐332 and type XVII collagen expression in reconstructed skins. (A) Immunostaining of Laminin‐332 in the obtained reconstructed skins. (B) Immunostaining of type XVII collagen in the obtained reconstructed skins. Quantification of (C) Laminin‐332 and (D) Type XVII collagen expression levels. Analysis was performed using three reconstructed models per condition of treatment and three representative pictures were taken by sample. For each picture, total positives red pixels were rationalized by dermal epidermal junction length. Student's test: *p < 0.05, **p < 0.01, ***p < 0.001.

Deposition of crucial basement membrane components that are significantly impacted during skin aging, such as laminin‐332 and type XVII collagen, was analyzed by immunostaining (Figure 4). As shown by representative microscopic pictures in Figure 4A, laminin‐332 was weakly expressed and often visualized as punctiform staining in the aged UT control. In striking contrast, the expression of laminin‐332 was highly enhanced (+209%, *p* < 0.001) in IPN‐12.5‐treated SE constructs, resulting in a regular and linear deposition at the DEJ level, thereby indicating more advanced tissue maturation, organization, and robustness. HA‐X 12 also increased laminin‐332 expression compared to the UT condition but to a lesser extent; however, this increase was still significant (57% (*p* < 0.001)) (Figure 4C).

Concomitant with early deposition of basement membrane components, IPN‐12.5 improved the epidermal stem cell reservoir, as demonstrated by enhanced expression of type XVII collagen around the basal keratinocytes (+ 53%, *p* < 0.001) compared to the UT control. However, type XVII collagen was not modulated by HA‐X 12 compared to the UT control (Figure 4B,D). These immunohistochemical observations were further confirmed by automated quantification and indicated a statistically significant increase in the expression of basement membrane components involved in the morphogenesis of the DEJ in IPN‐12.5 treated conditions (Figure 4C,D). Moreover, the number of proliferative Ki‐67 positive cells was significantly improved by 188% (*p* < 0.001) after IPN‐12.5 injections, unlike following HA‐X 12 treatments (data not shown).

### 3.4 Effect of IPN‐12.5 on Terminal Differentiation and Cellular Cohesion in Aging SE

**FIGURE 5:**
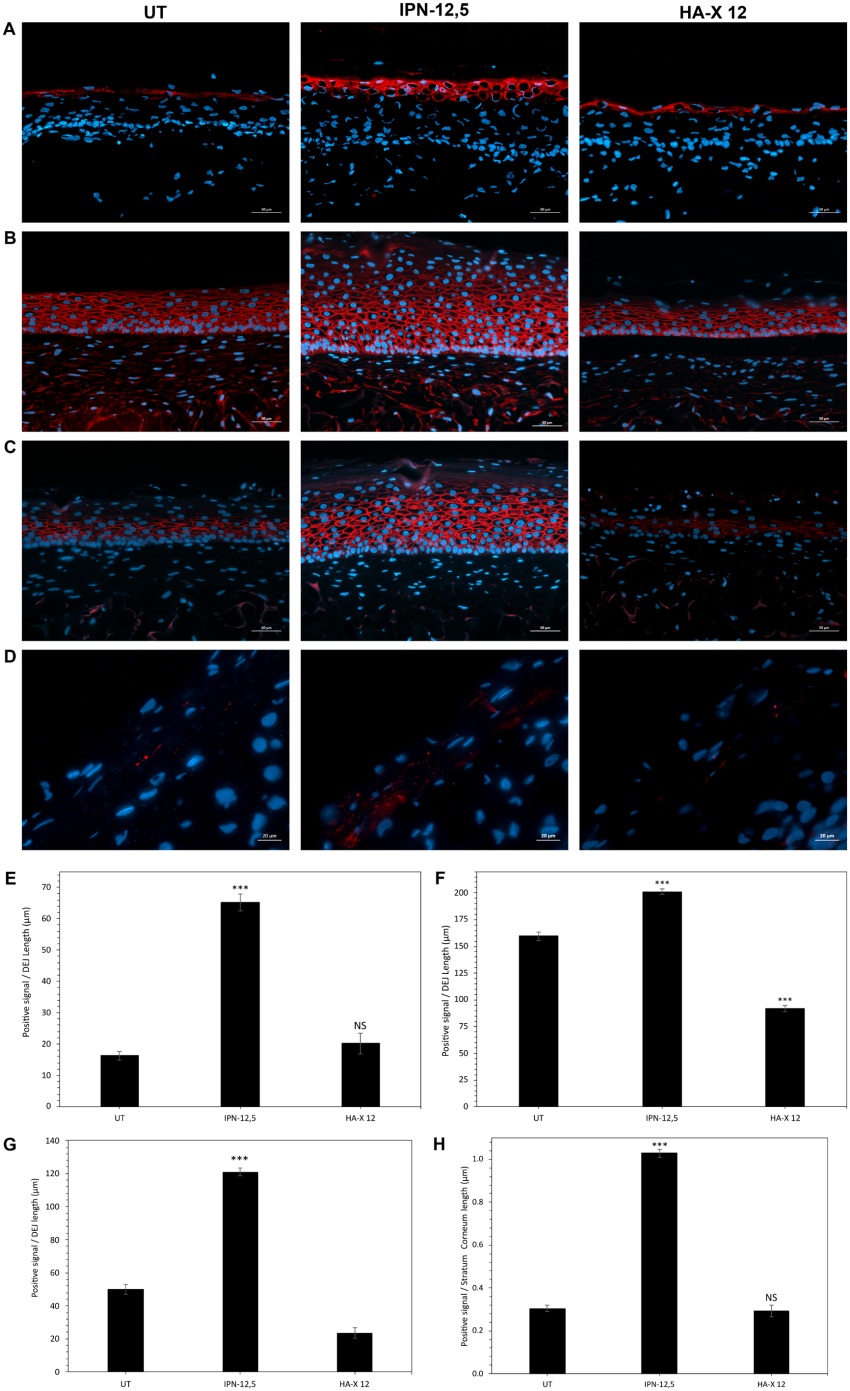
Effects of the two evaluated hyaluronic acid‐based hydrogels on filaggrin (A), CD44 (B), AQP3 (C), and ceramides (D) expression in reconstructed skins. Quantification of filaggrin (E), CD44 (F), AQP3 (G), and ceramides (H) expression levels. Analysis was performed using three reconstructed models per condition of treatment and three representative pictures were taken by sample. For each picture, total positives red pixels were rationalized by dermal epidermal junction length or regions of interest. Student's test: *p < 0.05, **p < 0.01, ***p < 0.001.

To evaluate epidermal homeostasis, late differentiation (filaggrin), lipid synthesis (ceramides), cellular cohesion (CD44), and tissue hydration (AQP3) were examined (Figure 5). Compared to the UT aged control, the IPN‐12.5 group exhibited a significant increase in the expression of filaggrin (+ 301%, *p* < 0.001, Figure 5A,E) and ceramides (+ 170%, *p* < 0.001; Figure 5D,H). In contrast, HA‐X 12 was not associated with similar changes in these epidermal‐related proteins. Moreover, IPN‐12.5‐injected SE models showed a highly enhanced expression of AQP3 compared to the untreated aged control (Figure 5C,G). There was also a significant increase in the expression of the hyaluronic acid‐specific CD44 receptor after IPN‐12.5 injections only (Figure 5B,F).

## 4 Discussion

Thirty years ago, 3D skin models began to be used as a prospective tool for wound covering drug testing in the context of severe burns [19] In vitro 3D cell culture methods are increasingly considered relevant tools to mimic in vivo conditions. Furthermore, 3D systems have been demonstrated to mimic living tissues in vivo in terms of bio‐structural and bio‐functional properties better than 2D culture techniques with regards to cellular functions [20]. The 3D SE used in this study was based on a scaffold, giving the advantage of yielding a genuine dermis‐type matrix, involving basement membrane structures and thereby allowing the study of epithelial–mesenchymal interactions in skin regeneration and epidermal formation [19]. Hence, the bespoke skin model was developed to meet these precise specifications. Indeed, it had to be “Full‐thickness” skin model and was crucial to include a dynamic dermal compartment, thereby allowing the injection of the investigated HA‐based products into the dermal component. The skin model also included an epidermis to allow for the study of protein targets involved in tissue hydration and barrier function. We also ensured that the culture time was long enough to perform several sessions of injections to closely replicate the recommended protocol in clinical practice for skin quality addressing treatment. Furthermore, we used a multi‐point injection technique in the skin model rather than bolus injections to enhance the clinical applicability of the study, again ensuring techniques closely resemble those used in clinical practice. Finally, the effects of both IPN‐12.5 and HA‐X 12 were characterized at the epidermal, dermal, and DEJ levels, and all compared to UT samples subjected to identical conditions aside from HA‐based gel to mitigate the risk of bias that could have been introduced through the physical stress of the actual injections.

Treatment with IPN‐12.5 hydrogel led to a statistically significant increase in epidermal thickness, with total AQP3 quantities significantly higher than in the UT condition (+142%, *p* < 0.001). The large quantities of this water and glycerol transporter seem to have led to a better closed water circuit in these samples and thus a better hydrated epidermis. Furthermore, these larger quantities of circulating water were probably efficiently retained in the samples because of an improved barrier function, which was demonstrated by the detection of filaggrin and ceramides in larger quantities following IPN‐12.5 injection compared to UT conditions. Although there was a significant improvement in TEWL following HA‐X 12 injections, we were not able to elucidate a clear biological mechanism to explain this observation. In 2002, Sougrat et al. [3] were the first to report that AQP3 distribution in the epidermis was consistent with epidermal water distribution and parallels the steep water gradient at the junction between the *stratum granulosum* and the *stratum corneum* (SC). It has been demonstrated that AQP3 expression decreases with aging and that it is associated with defective osmotic equilibrium in the epidermis, leading to skin dryness [4]. Moreover, phenotypic studies in transgenic mice lacking AQP3 confirmed the physiological importance of AQP3 in skin hydration, since knock‐out animals exhibited dry skin, reduced skin elasticity, delayed barrier recovery and wound healing, and also reduced glycerol content [21].

IPN‐12.5‐injected SE demonstrated improved homeostasis, evidenced by the significant increase in stem cells niche‐specific markers Ki67 and type XVII collagen as well as laminin 332 expression. These elements are known to play a critical role in restoring the equilibrium of aged skin, which allows recovery of all epidermal layers from the least to the more differentiated since they all play a critical role in skin health. It is also possible that the large quantities of the receptor CD44 and AQP3 detected in the epidermis, in response to IPN‐12.5 injection, could have stimulated the differentiation of keratinocytes, as previously described for both proteins [6]. Indeed, in keratinocyte culture, it has been shown that CD44 activation by HA upregulated the expression of keratinocyte differentiation markers, such as involucrin, pro‐filaggrin, and CK‐10. It has also been reported that aquaporins are one of the critical factors in the disruption of the skin barrier in several skin diseases [22]. All these changes support the role of IPN‐12.5 gel as a dermal injectable product acting on skin hydration.

Dermal fillers injections in vivo or into biopsies lead to an increase in type I collagen synthesis [23, 24]. From a mechanical perspective, it has been demonstrated that enhancing the structural support of the dermal ECM secondary to intradermal injection of HA‐based gels is associated with increased epidermal proliferation and thickening in aged skin.^23^ Dermal IPN‐12.5 injections also produced a significant increase in type 1 collagen and EMILIN‐1 content in both the papillary and reticular dermis, demonstrating improved skin firmness and elasticity, respectively. However, although HA‐X 12 was associated with an increase in filaggrin in the most differentiated epidermal layer and laminin‐332 in the DEJ, all the other tested markers had degraded expression levels. Therefore, the observed effects of HA‐X 12 on skin quality were a mixture of favorable and deleterious biological changes, hindering our ability to make clear conclusions on the product's skin hydration and elasticity improvement claims.

Even though both IPN‐12.5 and HA‐X 12 are commercialized hydrogels dedicated to similar indications, they seem to trigger distinctly different tissue reactions. IPN‐12.5 and HA‐X‐12 are two products derived from different technologies, particularly in the cross‐linking process. Additionally, even though their concentrations are similar, it is entirely possible that the molecular weight of the HA used in their manufacture is significantly different. High‐molecular‐weight hyaluronic acid (HMW) has long been known to inhibit endothelial cell proliferation [25]. Conversely, low‐molecular‐weight hyaluronic acid (LMW) has been shown to have an important role in wound healing and tissue remodeling processes [26]. To explain these differences, Pardue et al. [27] hypothesized that when LMW binds to its receptor (e.g., CD44), the small size of the ligand allows receptor clustering, which does not occur when HMW binds to the same receptor. As a result, intracellular signaling may differ greatly, leading to very distinct biological effects. Thus, HA biological effects could be different depending on the respective proportion of HMW and LMW between the two products. This hypothesis could be a potential explanation for the different observed effects of IPN‐12.5 and HA‐X 12.

One of the objectives of this study was mainly to elucidate the mechanisms of action of these two products by which means they managed to significantly improve the hydration and quality of the skin. Hypotheses were made on the impact of the size of the HA fragments present in the respective formulations. Future investigations concerning the characterization of the distribution of the size of the fragments in each of the two products and then the reiteration of this type of in vitro study by injecting certain fragment sizes of fragments could lead us to clarify the implications of each in the identified mechanisms. More generally, a future careful analysis of all HA‐X 12 formulation characteristics could allow us to identify or, at least, propose hypotheses to explain the observed differences.

Our study presents some limitations and strengths. Like any in vitro system, our model does not perfectly replicate the native tissue. Indeed, some cutaneous cell types are not present (melanocytes, endothelial, and immune cells). It is possible that the absence of immune cells in our model may underestimate the inflammatory response that modulates HA efficacy in vivo, or that the presence of a vascular network could add some crucial communication factors into the biological system and impact the expression of some of the targeted proteins. We should also highlight the fact that the dermo‐epidermal papillae are not present. Nevertheless, the skin model used in this study remains superior to models based on monolayer cultures, which do not allow the replication of injection conditions and can only be maintained in culture for a few days while retaining the correct cellular phenotype. Moreover, it is superior to ex vivo models, where cells in explants tend to quickly shift their focus to survival rather than performing their physiological role as discussed in the introduction of the manuscript [14]. For example, we demonstrated that when reconstructed skins were synthesized using cells from an elderly donor, the result accurately reflected the in vivo characteristics of aging with a thin epidermis demonstrating impaired terminal differentiation, a loose dermo‐epidermal junction, and sparse dermal extracellular matrix. We attempted to mitigate the potential bias that could result from the technique of injection by using a standardized protocol, one injector who used the same reference of short needles and used the needle's hub to master the dermal constant injection depth. Furthermore, to eliminate the effect of mechanical trauma related to the injection itself, we controlled for conditions that could have confounded cellular stimulation or skin hydration. Indeed, the use of a control allowed us to observe the effects attributable to the HA molecules present in the evaluated products independent of the physical trauma related to the injection or the water they contain. However, we did not perform a control “needle‐only” (no substance injected), making it difficult to fully separate the mechanical effects of injection from the ones related to the formulation, which can be considered a limitation to our work. Our future studies will incorporate a needle‐only control to enable us to isolate mechanical trauma impact. Finally, we did not have information about the detailed technology of the competitor product, and hence, it was not possible to explore any correlations between the specific characteristics of each individual product and the changes identified in the skin model.

The clinical interpretation of our results must be approached with contextual caution and within the research parameters outlined by the description of our skin model. However, we observed a 20% improvement in TEWL with the IPN‐12.5 treatment (Figure 2C). This parameter is a value typically measured in clinical settings when evaluating the moisturizing capacity of a product. Consequently, our results potentially reflect the underlying mechanisms involved in hydration improvement following the injection of a specific cross‐linked HA gel formulation.

## 5 Conclusion

The results of this study provide a better understanding of the action of cross‐linked hyaluronic acid gel when injected into the superficial layers of the skin, namely the dermis. Clinically, such treatments typically result in improved hydration and tissue quality. We now know that the enhancement of the water content, the contribution of which is inherent to this type of injection, is not the only factor to consider. The tissue's ability to utilize this water is reflected in the level of AQP3 expression. Similarly, the synthesis of NMF and the reinforcement of the skin barrier, which helps limit evaporation, may reduce water loss, as evidenced by the decrease in TEWL. This parameter is particularly important, as it is also commonly measured in clinical protocols. Thus, while acknowledging the limitations of our model, we can hypothesize that these mechanisms are also involved in vivo and can be potentially validated clinically. Finally, our study also revealed that subtle differences in the composition of products can result in significantly different levels of protein expression. However, the underlying mechanisms are still poorly understood. Hence, it will be important in the future to better understand these mechanisms so as to potentially maximize the clinical effects of products offered to patients to restore and treat skin quality as well as the potential long‐term outcomes of repeated injections.

## Author Contributions

M.A. Study rational, protocol design, manuscript drafting, and manuscript reviewing. K.L. Technical manipulations on the bench. M.D.S. Study protocol advisory. D.C. Study protocol advisory, manuscript reviewing. F.T. Study rational, study protocol advisory, manuscript reviewing, and head of the department.

## Ethics Statement

The authors confirm that the ethical policies of the journal, as noted on the journal's author guidelines page, have been adhered to and the appropriate ethical approvals have been received. Human skin tissue was collected according to the Declaration of Helsinki Principles and its use was declared to the French Research Ministry (declaration n° DC‐2020‐4346). A written informed consent was obtained from the donor according to the French bioethical law of 2014 (Law 94–954 of July 29, 1994). Primary cultures of human fibroblasts and keratinocytes were established from healthy skin biopsies obtained from an adult Caucasian donor (> 40 years old). Normal human epidermal keratinocytes (NHEK) and dermal fibroblasts (NHDF) were isolated from human skin as previously described.

## Consent

The authors have nothing to report.

## Conflicts of Interest

Marion Albouy, Denis Couchourel, and Frédéric Tranchepain are employed by Laboratoires VIVACY. Kilian Laho and Morgan Dos Santos are employed by LabSkin Creations, which was contracted by Laboratoires VIVACY to conduct this study.

## Data Availability

The data that support the findings of this study are available on request from the corresponding author. The data are not publicly available due to privacy or ethical restrictions.
